# Arabinoxylo- and Arabino-Oligosaccharides-Specific α-L-Arabinofuranosidase GH51 Isozymes from the Amylolytic Yeast *Saccharomycopsis fibuligera*

**DOI:** 10.4014/jmb.2012.12038

**Published:** 2021-01-01

**Authors:** Tae Hyeon Park, Chang-Yun Choi, Hyeon Jin Kim, Jeong-Rok Song, Damee Park, Hyun Ah Kang, Tae-Jip Kim

**Affiliations:** 1 Division of Animal, Horticultural and Food Sciences, Graduate School of Chungbuk National University, Cheongju 28644, Republic of Korea; 2 Department of Life Science, Chung-Ang University, Seoul 06974, Republic of Korea

**Keywords:** *Saccharomycopsis fibuligera*, α-L-arabinofuranosidases, arabino-oligosaccharides, arabinoxylooligosaccharides, L-arabinose

## Abstract

Two genes encoding probable α-L-arabinofuranosidase (E.C. 3.2.1.55) isozymes (ABFs) with 92.3% amino acid sequence identity, *ABF51A* and *ABF51B*, were found from chromosomes 3 and 5 of *Saccharomycopsis fibuligera* KJJ81, an amylolytic yeast isolated from Korean wheat-based nuruk, respectively. Each open reading frame consists of 1,551 nucleotides and encodes a protein of 517 amino acids with the molecular mass of approximately 59 kDa. These isozymes share approximately 49% amino acid sequence identity with eukaryotic ABFs from filamentous fungi. The corresponding genes were cloned, functionally expressed, and purified from *Escherichia coli*. SfABF51_A_ and SfABF51_B_ showed the highest activities on *p*-nitrophenyl arabinofuranoside at 40~45°C and pH 7.0 in sodium phosphate buffer and at 50°C and pH 6.0 in sodium acetate buffer, respectively. These *exo*-acting enzymes belonging to the glycoside hydrolase (GH) family 51 could hydrolyze arabinoxylo-oligosaccharides (AXOS) and arabino-oligosaccharides (AOS) to produce only L-arabinose, whereas they could hardly degrade any polymeric substrates including arabinans and arabinoxylans. The detailed product analyses revealed that both SfABF51 isozymes can catalyze the versatile hydrolysis of α-(1,2)-and α-(1,3)-L-arabinofuranosidic linkages of AXOS, and α-(1,2)-, α-(1,3)-, and α-(1,5)-linkages of linear and branched AOS. On the contrary, they have much lower activity against the α-(1,2)-and α-(1,3)-double-substituted substrates than the single-substituted ones. These hydrolases could potentially play important roles in the degradation and utilization of hemicellulosic biomass by *S. fibuligera*.

## Introduction

L-Arabinose is a pentose sugar that can be obtained by the hydrolysis of various plant cell wall hemicellulosic polymers such as arabinoxylans and arabinans [1]. Due to the health-beneficial functions, L-arabinose and arabino-oligosaccharides (AOS) have been considered as promising candidates for low-calorie sweeteners and prebiotic materials [2-4]. For the enzymatic production of L-arabinose, α-L-arabinofuranosidase (ABF; E.C. 3.2.1.55) is the key player which catalyzes the *exo*-type hydrolysis of L-arabinose-containing polymers [5, 6]. Especially, its synergistic actions with *endo*-α-(1,5)-L-arabinanase (ABN; E.C. 3.2.1.99) were suggested for the cost-effective L-arabinose production from sugar beet arabinan [7, 8].

The ABFs possess versatile hydrolyzing activities towards the terminal non-reducing α-(1,2)-, α-(1,3)-, and/or α-(1,5)-L-arabinofuranosidic linkages in various polymeric and oligomeric substrates [5, 6]. Depending on their structural features, the common microbial ABFs can be mainly categorized to the members of glycoside hydrolase (GH) families 51 and 43. To date, various ABFs have been reported from mainly bacteria, as well as a few fungi and plants [5, 6, 9]. Based on the genome analyses, the probable gene clusters for the utilization of L-arabinose and arabinans were found from *Bacillus* [10, 11], *Geobacillus* [12], and *Corynebacterium* spp. [13]. *Bacillus subtilis* produces two intracellular *exo*-ABFs GH51 and two extracellular *endo*-ABNs GH43, which can synergistically degrade the arabinan polymers to L-arabinose [11]. On the contrary, only a limited number of eukaryotic ABFs were genetically and enzymatically characterized from the fungal microorganisms such as *Penicillium* [14], *Aspergillus* [15], *Chrysosporium* [16], and *Aureobasidium* spp. [17]. As the eukaryotic ABFs share relatively low amino acid sequence identities with each other, it is very difficult to predict and categorize the functions of eukaryotic ABFs.

For the yeast ABFs, only a few enzymatic characteristics without any genetic basis were previously reported from *Rhodotorula* [18] and *Pichia* spp. [19]. The L-arabinose transport and catabolism were also investigated from yeasts such as *Candida* and *Pichia* spp. [20]. Recently, the genome and transcriptome analyses revealed that *Saccharomycopsis fibuligera* KJJ81 possesses two probable ABF genes located on the different chromosomes [21]. In addition to α-amylases and α-glucosidases, *S. fibuligera* was known to secrete various acid proteases and cellulose- and cell wall-degrading enzymes such as β-glucosidases and β-glucanases, which can be applicable for the fermentation industry [21, 22]. Especially, the yeast isolate KJJ81 was originated from wheat-based nuruk, the traditional Korean alcohol fermentation starter. Accordingly, this amylolytic yeast is supposed to produce additional hemicellose-degrading enzymes such as ABFs.

In the present study, the two genes encoding probable ABFs (hereafter abbreviated as SfABF51_A_ and SfABF51_B_) were first cloned from the amylolytic yeast *S. fibuligera* KJJ81, and their enzymatic properties were characterized in detail. Furthermore, the hydrolytic modes of action of these eukaryotic ABFs were comparatively investigated with the other known prokaryotic ABFs belonging to GH51 and GH43.

## Materials and Methods

### Enzyme Substrates and Oligosaccharide Standards

Sugar beet arabinan (SA), debranched arabinan (DA), arabinoxylans (AXs), arabino- and arabinoxylo-oligosaccharides (AOS and AXOS) were purchased from Megazyme (Ireland). The abbreviations of AOS and AXOS were summarized as A1~A6 (L-arabinose to arabinohexaose), AA^3^A (3^2^-α-L-arabinofuranosyl-arabinotriose), AAA^3^A (3^2^-α-L-arabinofuranosyl-arabinotetraose), AA^2+3^A (2^2^,3^2^-di-α-L-arabinofuranosyl-arabinotriose), A^3^X (3^2^-α-L-arabinofuranosyl-xylobiose), A^2+3^X (2^2^,3^2^-di-α-L-arabinofuranosyl-xylobiose), A^2^XX (2^3^-α-L-arabinofuranosyl-xylotriose), A^3^XX (3^3^-α-L-arabinofuranosyl-xylotriose), XA^2^XX (2^3^-α-L-arabinofuranosyl-xylotetraose), and XA^3^XX (3^3^-α-L-arabinofuranosyl-xylotetraose).

### cDNA Synthesis and RNA Sequencing Analysis

The amylolytic yeast *S. fibuligera* KJJ81 was isolated from Jeju Island in Korea [21]. For the cDNA synthesis of *S. fibuligera* KJJ81 *ABF51A* (KJJ81A3G090700.1) and *ABF51B* (KJJ81B5G075900.1), total RNA was prepared from the yeast cells grown exponentially using the glass bead-mediated breakage in a formamide/EDTA solution [23]. *S. fibuligera* cells were cultivated with shaking at 220 rpm in YPD broth (1% yeast extract, 2% peptone, and 2% glucose) at 37°C. The purified RNA sample (1 μg) was reacted with RnaUsScript Reverse Transcriptase (LeGene 6150, USA) at 45°C for 1 h. For the RNA-Seq analysis, the yeast cells were cultivated in YPD medium with 0.1% and 2.0% of glucose at 37°C for 12 h. From the grown cells, total RNA was isolated by the physical cell-breaking method, and the RNA-Seq analysis was performed using the Illumina HiSeq 2500 (USA) [21].

### Gene Cloning, Expression, and Enzyme Purification

*S. fibuligera* *ABF51* genes were amplified from the cDNA template with the primer sets for SfABF51_A_ (5’-TTTTCATATGACATCTTACAATCAAACTA-3’ and 5’-TTTTCTCGAGAGCGTAGGTGACTTCCAAA-3’) and SfABF51_B_ (5’-TTTTCATATGACATCTTACAATCAAACTA-3’ and 5’-TTTTCTCGAGAGCATAGG TGACTTGCAAAA-3’), respectively. PCR was performed with Pyrobest polymerase (Takara, Japan) using a C1000 Thermal Cycler (Bio-Rad, UK) as follows: 1 cycle of 98°C for 30 sec, 30 cycles of 98°C for 10 sec, 54°C for 30 sec, 72°C for 1 min 30 sec, and 1 cycle of 72°C for 5 min. The PCR fragments digested with NdeI and XhoI were cloned into pET-21a, and were designated as pESfABF51_A_ and pESfABF51_B_, respectively. *E. coli* BL21(DE3) harboring pESfABF51 was cultivated in LB broth containing 100 μg/ml ampicillin and 0.1 mM IPTG at 37°C for 14 h. *E. coli* cells were disrupted by ultrasonication (VCX750; Sonics & Materials, USA). The SfABF51 enzyme with C-terminal six histidines was purified by using AKTA Prime with a HisTrap FF column (GE Healthcare, Sweden). The expression level and purity of SfABF51 were examined using 12% SDS-PAGE analysis. The protein concentration was determined by using a BCA Protein Assay Kit (Pierce Biotechnology, USA).

### Enzyme Activity Assays for SfABF51

The activity towards *p*-nitrophenyl arabinofuranoside (*p*-NPAf; Sigma-Aldrich, USA) was determined by measuring the amount of *p*-nitrophenol being liberated by the enzymatic reaction. The L-Arabinose/D-Galactose Assay Kit (Megazyme) was utilized for the measurement of enzyme activity towards AOS and AXOS. The purified enzyme was reacted with 0.5% of each substrate under the optimal conditions for an appropriate reaction time. One unit of ABF activity was defined as the amount of enzyme to produce 1 μmol of L-arabinose equivalent from each substrate for 1 min.

### TLC Analysis for Hydrolysis Products

The enzymatic hydrolysis products were comparatively identified by using thin-layer chromatography (TLC). Under the optimal conditions, each substrate (0.5%) was reacted with an appropriate amount of enzyme for 15 h. The resulting hydrolysates were separated on a 60F_254_ silica gel glass plate (Merck, Germany) with a solvent of chloroform/acetate/water (6:7:1). The product spots were visualized and identified by dipping in the developing solution (0.3% *N*-1-naphthyl-ethylenediamine and 5% H_2_SO_4_ in methanol), followed by heating at 110°C for 10 min.

### 3D Structure Modeling of SfABF51 Isozymes

The primary structure comparison among microbial ABFs was performed by using CLC Main Workbench 20 (Qiagen, Denmark). Based on the amino acid sequence homology, three-dimensional structures of SfABF51 isozymes were predicted via the SWISS-MODEL (https://swissmodel.expasy.org), an automated protein structure homology-modeling server. The 3D structure models were visualized and drawn by using PyMOL software (DeLano, LLC).

## Results and Discussion

### Gene Cloning and Expression of SfABF51 Isozymes

**Fig. 1:**
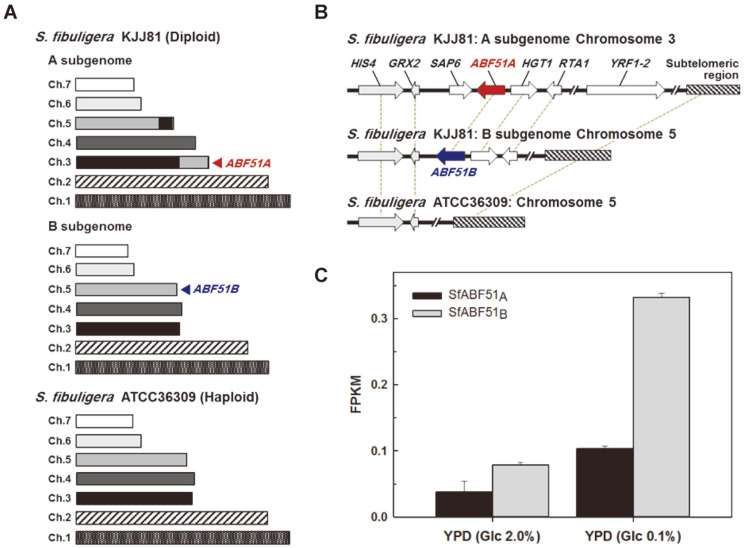
The gene clusters including *ABF51* in *S. fibuligera* chromosomes and the transcription levels of *ABF51* under different glucose concentrations. (**A**) The overall chromosome structures of *S. fibuligera* KJJ81 and ATCC36309. (**B**) The comparison of gene clusters including *SAP6, ABF51, HGT1, RTA1*, and *YRF1-2* found in chromosome 3 of *S. fibuligera* KJJ81 subgenome A. (**C**) Transcription analyses of two *ABF51* isogenes during the cultivation with YPD medium containing 0.1% and 2.0% glucose, respectively. The transcription levels of *ABF51* were statistically analyzed based on the previous RNASeq data [21]. FPKM, fragments per kilobase of exon model per million reads mapped.

The amylolytic yeast *S. fibuligera* KJJ81 was isolated from a nuruk sample in Korea [21]. The genome of *S. fibuligera* KJJ81 (~38 Mb) is a heterozygous diploid which consists of two subgenomes A and B sharing 88.1%nucleotide identity. The hybrid genome of *S. fibuligera* contains seven chromosome pairs (Fig. 1A). While searching for the hydrolytic enzyme genes, two homologs of *Aspergillus nidulans* *abfC* were previously found from *S. fibuligera* genome [21]. Due to the reciprocal translocation between chromosomes 3 and 5 in the subgenome A, two genes encoding SfABF51_A_ and SfABF51_B_ are present on two different chromosomes in the diploid genome of *S. fibuligera* KJJ81; one located on chromosome 3 in subgenome A and the other located on chromosome 5 in subgenome B. On the other hand, chromosome 5 of *S. fibuligera* ATCC36309, which was isolated from chalky rye bread in Germany, lacks the gene clusters including *ABF51* (*abfC*) at the subtelomeric regions of *S. fibuligera* KJJ81 chromosomes (Fig. 1B).

The previous RNA-Seq data showed that the expressions of both *ABF51* isogenes were highly induced under glucose-limited conditions [21]. These results indicated that the production of each ABF is subject to the catabolite repression at transcription levels in the presence of glucose. According to transcriptome analyses, it was verified that the transcription level of *ABF51B* is much higher than that of *ABF51A* (Fig. 1C). Such difference in the transcription levels might be influenced by the different locations of the two genes, which was caused by the rearrangement of chromosomes.

**Fig. 2:**
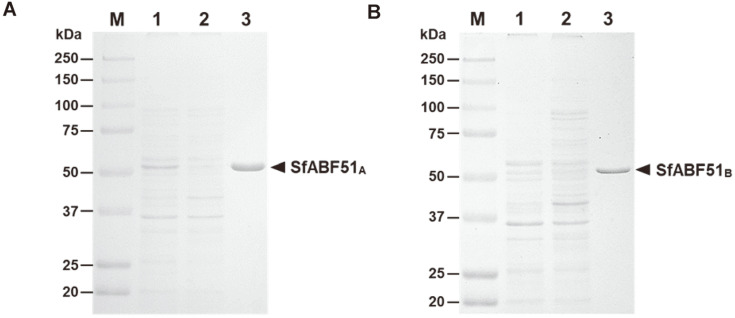
SDS-PAGE analysis for gene expression and enzyme purification of (A) SfABF51_A_ and (B) SfABF51_B_ from *E. coli*. Lane M, protein size marker; 1, cell extract from *E. coli* harboring pESfABF51; 2, flow-through fraction with unbound proteins; 3, recombinant SfABF51 purified by Ni-NTA chromatography.

The open reading frames of both *ABF51* isogenes consist of 1,551 nucleotides encoding the proteins of 517 amino acids (~59 kDa), sharing 92.3% amino acid sequence identity with each other (Fig. S1). The absence of signal peptide in both SfABF51 isozymes suggests that they are expressed as intracellular enzymes. After PCR amplification with cDNA template, the PCR fragments were cloned into an inducible expression vector of pET-21a, and were designated as pESfABF51_A_ and pESfABF51_B_, respectively. The genes encoding SfABF51 with C-terminal histidine tag were expressed from *E. coli* BL21(DE3). The recombinant SfABF51 isozymes were purified to an apparent homogeneity using Ni-NTA chromatography (Fig. 2). The apparent molecular mass of recombinant SfABF51 including six histidines is approximately 52~53 kDa.

### Optimal Reaction Conditions for SfABF51 Isozymes

**Fig. 3:**
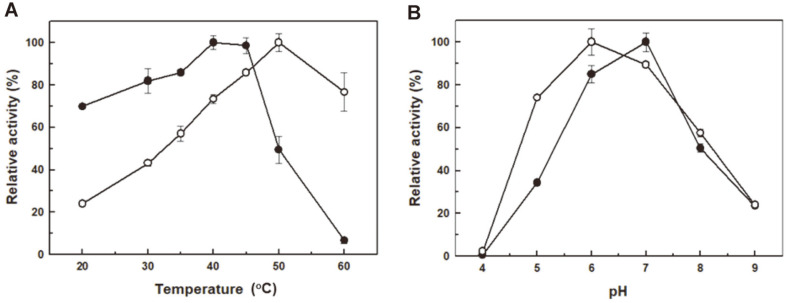
Effects of (**A**) temperature and (**B**) pH on enzyme activities of SfABF51 isozymes. Relative activities of SfABF51 isozymes on *p*-nitrophenyl arabinofuranoside at different temperatures and pH were measured at 405 nm. Sodium acetate (pH 4.0~6.0), sodium phosphate (pH 7.0), and Tris-HCl (pH 8.0~9.0) buffers were used for the activity assays.

The hydrolyzing activities of SfABF51 on various substrates were measured using *p*-nitrophenyl arabinofuranoside (*p*-NPAf) or L-arabinose assay. SfABF51_A_ exhibited the highest activity on *p*-NPAf (51.1 U/mg) in 50 mM sodium phosphate (pH 7.0) at 40~45°C, whereas SfABF51_B_ has optimum activity in sodium acetate (pH 6.0) at 50°C (Fig. 3). Interestingly, SfABF51_A_ and SfABF51_B_ showed significantly high activities (~70% of each optimum) at 20°C and 60°C, respectively. Common prokaryotic ABFs showed the highest activities at pH 5.0~8.0 and 50~70°C, whereas the fungal ABFs showed relatively lower optimal pH 3.0~5.0 and temperature of 40~70°C than prokaryotic enzymes [6]. The highly thermostable ABFs being active at 80~90°C were also reported from hyperthermophiles such as *Caldicellulosiruptor* [7] and *Thermotoga* spp. [24]. The yeast ABF from *Pichia capsulata* has slightly lower optimal pH 4.5, but a much higher optimum temperature at 75°C than those of SfABF51 isozymes. Meanwhile, *Rhodotorula flava* ABF showed the highest activity under the extraordinary conditions of pH 2.0 and 30°C [18].

### Hydrolytic Modes of Action of SfABF51 Isozymes

**Fig. 4:**
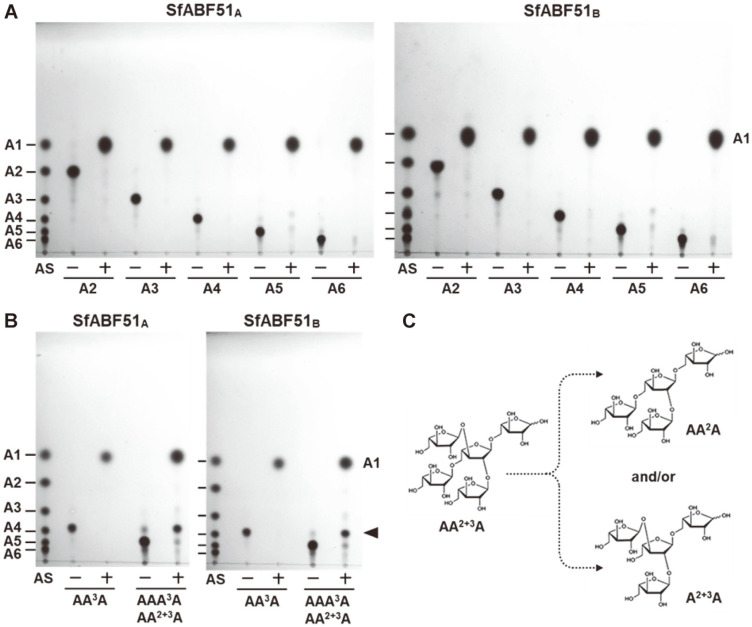
TLC analyses of the enzymatic hydrolysates from various linear and branched AOS reacted with SfABF51 isozymes. (**A**) LAOS or (**B**) BAOS substrate (0.5%) was reacted with 2.5 U/ml of SfABF51 isozymes at 37°C and each optimal pH for 15 h. The arrowhead indicates unidentified hydrolyzed product. (**C**) The predicted hydrolytic action modes of SfABF51 isozymes on AA^2+3^A. AS, AOS standards; A1~A6, L-arabinose to arabinohexaose; AA^3^A (3^2^-α-L-arabinofuranosylarabinotriose); AAA^3^A, 3^2^-α-L-arabinofuranosyl-arabinotetraose; AA^2+3^A, 2^2^,3^2^-di-α-L- arabinofuranosyl-arabinotriose.

To examine the hydrolysis patterns of SfABF51 isozymes, 2.5 U/ml of enzyme was reacted with 0.5% of each LAOS for 15 h, and the hydrolyzed products were comparatively identified by TLC analysis. As shown in Fig. 4A, both SfABF51 isozymes could exclusively produce L-arabinose from LAOS DP2~6. However, these enzymes showed no detectable activities on the polymeric substrates such as sugar beet (branched) and debranched arabinans. These results implied that SfABF51 can hydrolyze α-(1,5)-L-arabinofuranosidic linkages of LAOS, but not debranched arabinan polymer. When the BAOS was reacted with SfABF51, AA^3^A was completely hydrolyzed into L-arabinose, whereas the BAOS DP5 mixture of AAA^3^A and AA^2+3^A was partially degraded to L-arabinose and the unidentified BAOS DP4 (the arrowhead in Fig. 4B). As AA^3^A could be converted to L-arabinose, the partially hydrolyzed products might be A^2+3^A and/or AA^2^A (Fig. 4C). Accordingly, SfABF51_A_ and SfABF51_B_ are similar *exo*-type hydrolases that preferentially cleave the single-substituted α-(1,3)-L-arabinofuranosidic linkages of BAOS, whereas they showed very low activities on the double-substituted BAOS.

**Fig. 5:**
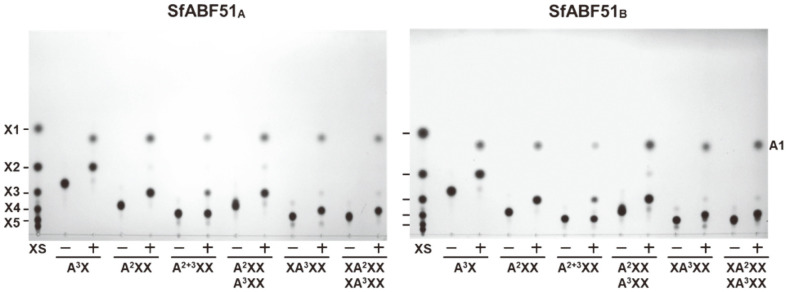
TLC analyses of the enzymatic hydrolysates from various AXOS reacted with SfABF51 isozymes. AXOS substrate (0.5%) was reacted with 1.5 U/ml of SfABF51 isozymes at 37°C and each optimal pH for 15 h. XS, XOS standards; X1~X5, D-xylose to xylopentaose; A^3^X, 3^2^-α-L-arabinofuranosyl-xylobiose; A^2+3^X, 2^2^,3^2^-di-α-L-arabinofuranosylxylobiose; A^2^XX, 2^3^-α-L-arabinofuranosyl-xylotriose; A^3^XX, 3^3^-α-L-arabinofuranosyl-xylotriose; XA^2^XX, 2^3^-α-L-arabinofuranosylxylotetraose; XA^3^XX, 3^3^-α-L-arabinofuranosyl-xylotetraose.

When 1.5 U/ml of SfABF51 was reacted with 0.5% AXOS for 15 h, most of the AXOS were completely debranched to linear XOS backbone and L-arabinose except for the double-substituted AXOS, A^2+3^XX (Fig. 5). As the double-substituted A^2+3^XX is resistant to the enzymatic hydrolysis by SfABF51 isozymes, most of the A^2+3^XX remained unhydrolyzed. These results revealed that both SfABF51 isozymes possess the debranching activities hydrolyzing α-(1,2)- or α-(1,3)-L-arabinofuranosyl branches, but not β-(1,4)-D-xylopyranosyl backbone of AXOS.

To understand the detailed modes of action, the specific activities of SfABF51 isozymes were investigated on various arabinose-containing polymers and oligomers (Table 1). Both SfABF51 isozymes could hardly hydrolyze any polymeric substrates such as arabinans and arabinoxylans, while they showed much higher hydrolyzing activities on oligomeric substrates including AOS and AXOS. Against most substrates, SfABF51_B_ exhibited 1.4~2.9 times higher specific activities than those of SfABF51_A_. The significantly high activities of SfABF51_A_ (51.1 U/mg) and SfABF51_B_ (75.7 U/mg) towards the synthetic substrate, *p*-NPAf, demonstrated that these enzymes are typical *exo*-acting arabinosyl hydrolases. Except for *p*-NPAf, SfABF51_A_ and SfABF51_B_ showed the highest activities (41.1 and 97.7 U/mg) on A^3^X, the shortest-chain AXOS, respectively. Especially, their activities on A^3^X (DP3) were 11.3~19.1 times higher than those on AOS (DP3) such as arabinotriose (A3) and A^3^A. Their specific activities on A^3^X (DP3) were 1.2~1.8 times higher than those on the longer AXOS (DP4) such as A^2^XX and A^2^XX+A^3^XX. In case of AOS, both SfABF51 isozymes showed the highest activities on the shortest-chain AOS, arabinobiose (A2), and were 5.5~31.7 times higher than the other longer LAOS and BAOS (DP3~6). As with the other common *exo*-hydrolases, it was observed that SfABF51 isozymes have significantly high preference for the shorter substrates compared to the longer ones. In addition, SfABF51 isozymes showed 1.2~1.3 times higher activities on the mixture of XA^2^XX and XA^3^XX than those on XA^3^XX. These results proposed that SfABF51_A_ and SfABF51_B_ preferentially hydrolyze α-(1,2)-L-arabinofuranosidic linkages in the internal branches of AXOS.

### Structural and Functional Relationship of SfABF51

**Fig. 6:**
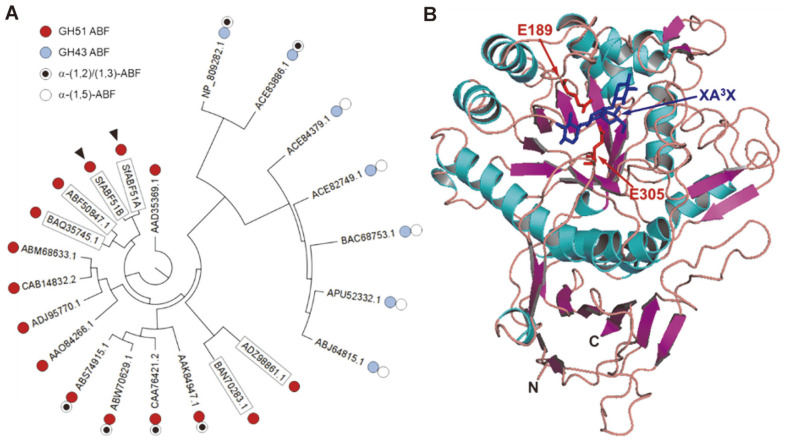
The primary and tertiary structure comparison among various microbial ABFs. (**A**) The phylogenetic tree among microbial ABFs was constructed through the neighbor-joining method. The eukaryotic ABFs are shown in boxes, and both SfABF51 isozymes are indicated by the arrowheads. The amino acid sequence IDs are summarized as the microbial ABFs from *Thermotoga maritima* (AAD35369.1), *Lactobacillus brevis* (ADJ95770.1, ABJ64815.1), *Bacillus substilis* (CAB14832.2, ABW70629.1), *B. velezensis* (ABS74915.1), *Geobacillus* sp. (ABM68633.1), *Bifidobacterium longum* (AAO84266.1), *Bacteroides thetaiotaomicron* (NP_809282.1), *Cellvibrio japonicus* (ACE82749.1, ACE83886.1, ACE84379.1), *Streptomyces* sp. (BAC68753.1), *Weissella* sp. (APU52332.1), *Pseudomonas cellulosa* (AAK84947.1), *Thermobacillus xylanilyticus* (CAA76421.2), *Aureobasidium pullulans* (BAN70283.1), *Aspergillus nidulans* (ABF50847.1), *Penicillium chrysogenum* (BAQ35745.1), and *Chrysosporium lucknowense* (ADZ98861.1). (**B**) The 3D structure model of SfABF51_A_ was predicted by SWISS-MODEL server. The (β/α)_8_- barrel catalytic domain contains two catalytic amino acid residues (red) with an AXOS ligand of XA^3^X (3^2^-α-L-arabinofuranosylxylotriose; blue).

Based on the amino acid sequences of the known microbial ABFs, the phylogenetic tree was drawn as a circular phylogram (Fig. 6A). SfABF51 isozymes showed the highest amino acid sequence identity (92.3%) with each other, and were closely related to the ABFs from *Penicillium chrysogenum* (BAQ35745.1) and *Aspergillus nidulans* (*abfC*, ABF50847.1).

The group of ABFs GH51 showing 23.9~25.9% amino acid sequence identity possess unique debranching activities on α-(1,2)- and/or α-(1,3)-L-arabinofuranosidic linkages, but not on α-(1,5)- linkages. *Bacillus velezensis* ABF can specifically hydrolyze α-(1,2)- and/or α-(1,3)-branches of sugar beet arabinan, BAOS, and AXOS, but it showed much lower activity towards α-(1,5)-linkages of short-chain LAOS [25]. Similar debranching modes of action were also reported on the other bacterial ABFs from *Bacillus subtilis* [11], *Pseudomonas cellulosa* [26], and *Thermobacillus xylanilyticus* [27]. Meanwhile, SfABF51_A_ showed high sequence identity (~49.0%) with the eukaryotic ABFs from the filamentous fungi, *P. chrysogenum* [14] and *A. nidulans* [15]. On the contrary, the other fungal ABFs from *Chrysosporium lucknowense* [16] and *Aureobasidium pullulans* [17] share very low sequence identity (13.8% and 12.4%) with SfABF51_A_, respectively. SfABF51_A_ showed 40.2% identity with *Thermotoga maritima* ABF [24] followed by the ABFs GH51 from *Geobacillus* sp. (31.4%) [8], *Bacillus subtilis* [11], and *Lactobacillus brevis* (30.2%) [28], and *Bifidobacterium longum* (26.5%) [29]. These prokaryotic ABFs are known to be members of GH51 which hydrolyze α-(1,2)-, α-(1,3)-, and/or α-(1,5)-L-arabinofuranosidic linkages of various arabinose-containing substrates. However, *Geobacillus* sp. ABF GH51 can hydrolyze both oligomeric and polymeric substrates, whereas SfABF51 isozymes showed no detectable activities on the latter.

On the other hand, the prokaryotic ABFs belonging to GH43 showed much lower amino acid sequence identity (< 6.5%) with SfABF51 isozymes than the other ABFs. Among those ABFs, *Bacteroides thetaiotaomicron* and *Cellvibrio japonicus* ABFs GH43 were reported to have α-(1,2)- and/or α-(1,3)-debranching activities [30]. The other α-(1,5)-L-arabinofuranosidic linkage-specific ABFs GH43 were originated from *Lactobacillus brevis* [31], *Cellvibrio japonicus* [30], *Streptomyces avermitilis* [32], and *Weissella* sp. [33]. These ABFs GH43 cannot hydrolyze any branched substrates such as BAOS and sugar beet arabinan.

Both SfABF51 isozymes share similar amino acid sequences and hydrolytic modes of action with the common prokaryotic ABFs belonging to GH51. As shown in Fig. 6B, the three-dimensional structure modeling of SfABF51 isozymes using the SWISS-MODEL server has revealed that the probable structures of SfABF51 were similarly predicted as the common structural architectures of ABFs GH51 consisting of the catalytic (β/α)_8_-barrel domain with C-terminal jelly-roll domain [24, 34]. Especially, the catalytic residues of *Thermotoga maritima* and *Thermobacillus xylanilyticus* ABFs were highly conserved at Glu189 and Glu305 in the model structure of SfABF51_A_ in complex with an AXOS ligand (XA^3^X). In conclusion, SfABF51_A_ and SfABF51_B_ possess primary and tertiary structures closely related to the prokaryotic ABFs GH51, as well as having similar hydrolytic modes of action.

Compared with *S. fibuligera* ATCC36309 isolated from chalky rye bread in Germany, *S. fibuligera* KJJ81 from wheat-based nuruk in Korea possesses two extra isogenes encoding functional SfABF51 which can be expressed under limited-glucose condition. In the present study, SfABF51_A_ and SfABF51_B_ were functionally characterized as *exo*-hydrolases having versatile hydrolyzing activities towards α-(1,2)-, α-(1,3)-, and/or α-(1,5)-L- arabinofuranosidic linkages of AOS and AXOS, but not towards polymeric substrates such as arabinans and arabinoxylans. Besides the degradation of hemicellulosic biomass, the ABFs from yeast and filamentous fungi have drawn additional attention for enhancing aromas in wine with their ability to hydrolyze grape monoterpenyl glycosides [35]. Therefore, the presence of two functional *ABF51* genes might be another attractive feature of *S. fibuligera* KJJ81 as an additional fermentation starter to provide diverse flavors for various types of fermented alcoholic beverages. The *ABF51* genes can be valuable genetic resources for developing genetically engineered yeast strains to produce flavor compounds at high levels. These findings are expected to help scientists better understand the carbohydrate utilization system of the amylolytic and hemicelluolytic yeast *S. fibuligera* and its applications in microbiology and biotechnology.

## Supplemental Materials



Supplementary data for this paper are available on-line only at http://jmb.or.kr.

## Figures and Tables

**Table 1:** Specific and relative activities of SfABF51 isozymes on various oligomeric substrates.

Type	Substrate	Specific activity (U/mg)^a^	

		SfABF51_A_	SfABF51_B_
Synthetic	*p*-NPAf	51.1±0.9 (100.0%)	75.7±4.1 (100.0%)

LAOS	A2	24.1±0.6 (47.2%)	70.4±3.8 (93.0%)
	A3	3.4±0.4 (6.7%)	5.5±0.4 (7.2%)
	A6	2.3±0.1 (4.5%)	2.2±0.2 (2.9%)

BAOS	A^3^A	3.6±0.1 (7.1%)	5.1±0.2 (6.8%)
	AA^3^A	4.4±0.1 (8.5%)	6.5±0.2 (8.5%)

AXOS	A^3^X	41.1±1.2 (80.5%)	97.7±7.0 (129.1%)
	A^2^XX	23.2±0.4 (45.4%)	55.0±1.9 (72.7%)
	A^2^XX+A^3^XX	31.3±0.9 (61.2%)	80.3±2.2 (106.1%)
	XA^3^XX	5.5±0.3 (10.8%)	12.8±0.3 (16.9%)
	XA^2^XX+XA^3^XX	7.31±0.19 (14.3%)	15.79±0.18 (20.9%)

^a^SfABF51 isozymes showed no detectable activities on any polymeric substrates including arabinoxylans and arabinans.
