# Supplementary material for: Arabinoxylo- and Arabino-Oligosaccharides-Specific α-L-Arabinofuranosidase GH51 Isozymes from the Amylolytic Yeast Saccharomycopsis fibuligera
Source: J Microbiol Biotechnol. 2021 Jan 1;31(2):272–9. doi: 10.4014/jmb.2012.12038 (PMC9705838; doi:10.4014/jmb.2012.12038)
Supplement: Supplementary file 1 [file jmb-31-2-272-supple.pdf]

J. Microbiol. Biotechnol.
https://doi.org/10.4014/jmb.2012.12038

Supplementary data

```
                      *        20         *        40         *        60         *
SfABF51A : MTSYNQTITEVESLHQSRIVVDPRRKLAPINPNIYSSFLEHLGRVIYGGIVDYNDPPSAPVNEQGFRTDV :  70
SfABF51B : MTSYNQTIREVESLHQSRIVVDPRRKLAPINPNIYSSFLEHLGRVIYGGIVDYAKPPSAPVNEHGFRIDV :  70

                 80         *       100         *       120         *       140
SfABF51A : SKAIQDLNVPVVRWPGGNYVSSYHWQDGVGPTENRPRRPELAWNGEESNLFGTDEFIEWCRYHKVEPYIC : 140
SfABF51B : SKAIQDLNVPVVRWPGGNYVSSYHWQDGIGPRENRPRRPELAWSGEESNLFGTDEFIEWCRYQKIEPYIC : 140

                      *       160         *       180         *       200         *
SfABF51A : LNMGTGTLDEALAWVEYCNSNANTYYANLRRANGHTEPYNVKYWGLGNEVWGDWQVGQQTAEDYAKNADQ : 210
SfABF51B : LNMGTGTLDEALAWVEYCNSNANTYYANLRRANGHPEPYNVKYWGLGNEVWGDWQVGQQTAEDYAKNADQ : 210

                220         *       240         *       260         *       280
SfABF51A : WGKAIKLLDPNIVLVSCGKTGVDNWDYHVLNQLINRVDLHSIHIYTASDSYIKNVTAPAAAEAAIQVTKN : 280
SfABF51B : WGKAIKLLDPNVVLVSCGKTGVDSWDYHVLDQLINRVDLHSIHIYTASDSYIKNVTAPAAAEAAIQITKN : 280

                      *       300         *       320         *       340         *
SfABF51A : LIDLATIQNSALANGKEKVKICFDEWNVWDPARADASKGLEEQYTLSDALAVASWLNVFVRQAETLAMCN : 350
SfABF51B : LIDLATIQHSALDNGKEKVKICFDEWNVWDPARAGASKGLEEQYTLSDALAVASWLNVFVRQAETLAMCN : 350

                360         *       380         *       400         *       420
SfABF51A : LAQLVNAIAPIVTSDSDLFLQSIYYPIQLFSKYMRNGYALNLHVDSTLYTGETGNNDGSYTWIQGNLQVP : 420
SfABF51B : LAQLVNAIAPIVTSKSDLFLQTIYYPLQLFSKYMRNGYSLNLHVDSTLYTGETGNNDGSYTWIQGNLRVP : 420

                      *       440         *       460         *       480         *
SfABF51A : LLDASAVQNNEDTKTYIAVVNRDENEDAFAKIAFTRKVRKIHQWHLYNDDVFAYNTIEKKETIALTEADL : 490
SfABF51B : LLDASAVQNDKDSKTYIAVVNRDENEDAFAKVAFPSKVRKIYQWHLYNDNVFAYNTIENKENIALTETDL : 490

                500         *
SfABF51A : EFQQDENGNIEILFKKHSFTFLEVTYA : 517
SfABF51B : DFEQDENNNVEIFFKKHSFTFLQVTYA : 517
```

Fig. S1. Comparison of amino acid sequences between SfABF51$_A$ and SfABF51$_B$. The amino acid sequence alignment was performed using GeneDoc software. The arrowheads indicate two catalytic residues (Glu189 and Glu305) of SfABF51 isozymes.
